# Decreased expression of hyaluronan synthase and loss of hyaluronan-rich cells in the anterior tibial fascia of the rat model of chemotherapy-induced peripheral neuropathy

**DOI:** 10.1097/PR9.0000000000001088

**Published:** 2023-06-27

**Authors:** Ruilin Wang, Yoshikazu Matsuoka, Nobutaka Sue, Kosuke Nakatsuka, Chika Tsuboi, Hiroshi Morimatsu

**Affiliations:** Department of Anesthesiology and Resuscitology, Okayama University Graduate School of Medicine, Dentistry and Pharmaceutical Sciences, Okayama, Japan

**Keywords:** Chemotherapy-induced peripheral neuropathy, Fascia, Fasciacyte, Hyaluronic acid, Musculoskeletal pain, Neuropathic pain

## Abstract

**Introduction:**

Previous studies on chemotherapy-induced peripheral neuropathy (CIPN) have focused on neuronal damage. Although some studies have revealed that the fascia is an important sensory organ, currently, we do not know about chemotherapy drug-induced fascial dysfunction.

**Objectives:**

This study aimed to explore the fascia as a nonneural cause of mechanical hypersensitivity in CIPN by investigating the expression of hyaluronic acid synthase (HAS) and histology of the fascia in an animal model of CIPN.

**Methods:**

Rats were intraperitoneally administered with vincristine (VCR). Mechanical hypersensitivities of the hind paw and the anterior tibial muscle were assessed. The expression of HAS mRNA in the fascia of the anterior tibial muscles was quantitated using reverse transcription polymerase chain reaction. Immunohistochemistry was also performed for HAS2, hyaluronic acid–binding protein, and S100A4 in the fascia.

**Results:**

Vincristine administration significantly decreased mechanical withdrawal thresholds in the hind paw and the anterior tibial muscle after day 3. Quantitative polymerase chain reaction showed significant downregulation of HAS mRNAs in the fascia of VCR-treated rats. Immunohistochemical analysis showed that the number of cells with strong HAS2 immunoreactivity, classified as fasciacytes by morphology and colocalized marker S100A4, decreased significantly in the VCR group.

**Conclusion:**

Hyaluronic acid plays a critical role in somatic pain sensation. Damaged fascia could be a possible cause of musculoskeletal pain in patients with CIPN. This study suggests that fascia is a nonneural cause and novel therapeutic target for chemotherapy-induced “peripheral neuropathy.”

## Abstract 2

Intraperitoneal vincristine administration decreased hyaluronan synthase and the number of hyaluronan-rich cells in the rat fascia, along with myofascial mechanical hypersensitivity in the lower leg.

## 1. Introduction

Chemotherapy-induced peripheral neuropathy (CIPN) is a common complication resulting from numerous commonly used anticancer drugs, such as paclitaxel, oxaliplatin, and vincristine (VCR).^11^ Up to 40 to 90% of patients with cancer reported symptoms of peripheral neuropathy during chemotherapy.^14,48^ Symptoms are mostly sensory, such as pain, altered touch sensation, numbness, and tingling.^48,53^ Although the frequency is low, motor symptoms, such as weakness and cramps, are observed.^48^ Currently, the pathogenesis of CIPN is recognized as axon and myelin damage, as well as neuronal lesions that occur through the cell bodies involved in the dorsal root ganglia.^30^ Based on this hypothesis, some drugs currently used for the treatment of neuropathic pain, such as amitriptyline, gabapentin, and duloxetine, have been prescribed for the treatment of CIPN. However, they have little analgesic effect in patients with CIPN, and even no effect, as some results reported in clinical randomized controlled trials.^19,34,38,40^

Fascia has long been considered solely as a connective tissue or supportive tissue that connects organs,^6^ as well as an organ or system that is often overlooked in pain research.^46^ In addition to its biomechanical properties, many recent studies have reported that it plays an important role in skeletal muscle movement, pain perception, protein regulation and expression, cell signaling, tumor growth, and hormone distribution.^33^ Recently, the fascia has been reported to contain dense free nerve endings and act as a sensory organ for nociception.^47^ There was also experimental evidence from animal and human trials that injecting an algogenic substance into the fascia is more painful than injecting it into the skin or muscle.^12,21,35^ However, the possible involvement of the fascia during chemotherapy is completely unknown.

Hyaluronic acid (HA), also known as hyaluronan, is a high-molecular-weight long-chain (5000−20,000,000 Da) unbranched polysaccharide composed of repeating disaccharides of d-glucuronic acid and N-acetyl-d-glucosamine.^36^ Hyaluronic acid is a major component of the extracellular matrix (ECM) that plays an important role in anti-inflammatory, analgesic, angiogenesis, cell migration, immune regulation, and homeostasis.^1,8^ It is synthesized by hyaluronan synthase (HAS) and extruded through the membrane into the extracellular space.^18^ Among the 3 mammalian isoforms, HAS1, 2, and 3, HAS2 comprises the majority.^4^ Hyaluronic acid is also abundant in the fascia, and several studies have shown that HA contributes to the maintenance of smooth gliding in the muscle and influences tissue viscosity.^45^ Recently, it has also been reported that the expression of HAS2 mRNA by fasciacytes, a novel class of fibroblasts in the fascia, is specialized to produce HA-rich ECM found in the connective tissue continuum.^44^ This study aimed to investigate the expression of HAS and fasciacytes in the fascia of an animal model of CIPN and to explore the fascia as a nonneural cause of the mechanical hypersensitivity observed in CIPN.

## 2. Methods

### 2.1. Animals

This study was approved by the Animal Care and Use Committee of Okayama University School of Medicine (OKU- 2020432). The animals were handled following the Ethical Guidelines for the Investigation of Pain in Conscious Animal Experiments published by the International Association for the Study of Pain.^54^

In this study, a total of 38 (30 for behavioral, 15 for polymerase chain reaction [PCR], and 16 for histological study) male Sprague-Dawley rats (CLEA Japan Inc, Tokyo, Japan) weighing 180 to 250 g at the time of administration were used. The animals were housed on soft bedding in plastic cages and provided food and water ad libitum. The room temperature was 24 ± 1°C, the humidity was 50 to 60%, and the light–dark cycle was 12 hours. The animals were randomly divided into different experimental groups.

### 2.2. Rat model of chemotherapy-induced peripheral neuropathy

As described previously,^20^ rats in the CIPN group were injected intraperitoneally with 0.1 or 0.2 mg/kg of VCR (Nippon Kayaku, Tokyo, Japan). The control rats were injected with 0.2 mL of normal saline.

### 2.3. Behavioral assessment

#### 2.3.1. Mechanical threshold in the hind paw

Pain behavior was evaluated before and 3, 5, and 7 days after administration. Mechanical allodynia was measured using the hind paw withdrawal threshold (PWT) with von Frey filaments (Touch-Test Sensory Evaluator; North Coast Medical, Morgan Hill, CA). Rats were placed individually in a plastic cage (13 × 10 × 15 cm^3^) with an elevated wire mesh bottom (opening, 5 × 5 mm^2^), allowing full access to the plantar surfaces of both hind paws. Mechanical stimuli were applied to the plantar aspect of each hind paw with 1 of the 9 von Frey filaments (0.4, 0.6, 1.0, 1.4, 2.0, 4.0, 6.0, 8.0, and 15.0 g). Each trial started with a von Frey force of 2 g for 1−2 seconds. The stimuli were given at intervals of at least several seconds, allowing for the apparent resolution of any behavioral responses to previous stimuli. Based on the response pattern and force of the final filament, the 50% PWT was determined by the up–down method^13^ and calculated using the formula described previously.^10^ If the strongest filament did not elicit a response, the PWT was recorded as 15.0 g.

#### 2.3.2. Mechanical threshold in the tibialis anterior muscle

For the assessment of muscle nociceptive hypersensitivity, an electronic von Frey aesthesiometer (IITC Life Science Inc, Woodland Hills, CA) was used to determine the withdrawal threshold to pressure stimuli as described previously.^22^ In brief, the torso of rat was secured with a cloth rolled to keep them calm. The rat was handled gently during the test and their legs could move freely. A blunt push rod with a flat surface (2.8 mm in diameter) to avoid skin injury was applied over the anterior side muscles of the lower legs. The withdrawal threshold referred to the pressure intensity inducing the leg reduction response. The test was repeated 4 times at the interval of several minutes and averaged. Training sessions were conducted every day for 5 days before the VCR administration. The thresholds were evaluated before and 3, 5, and 7 days after the administration.

### 2.4. Quantitative reverse transcription polymerase chain reaction

**Figure 1.**
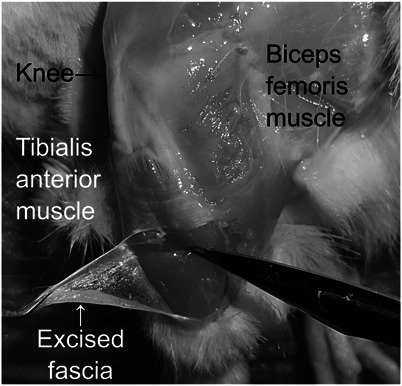
Handling of the anterior tibial fascia of the rat. After removal of the skin and subcutaneous connective tissue, the crural fascia covering the anterior tibial muscle is visible.

The anterior tibial fascia of the lower leg plays an essential role in the pain of delayed muscle soreness.^21^ Therefore, we used these samples in our study (Fig. 1). The rats were killed by decapitation under deep anesthesia. The bilateral anterior tibial fascia of the lower legs was removed and immediately stored in RNAprotect Tissue Reagent (Qiagen, Hilden, Germany). Total RNA was extracted using QIAzol Lysis Reagent (Qiagen) and RNeasy Lipid Tissue Mini Kit (Qiagen) according to the manufacturer's instructions. cDNA was synthesized using a QuantiTect Reverse Transcription Kit (Qiagen), according to the manufacturer's protocol. Genomic DNA was removed using the gDNA wipeout buffer included in the kit. Subsequently, quantitative PCR was performed using the StepOnePlus Real-Time PCR system (Applied Biosystems, Waltham, MA) and TB Green Premix Ex Taq II (Takara Bio, Shiga, Japan) at an annealing temperature of 60°C. The expression of rat HAS1, HAS2, HAS3, and glyceraldehyde-3- phosphate dehydrogenase (GAPDH) cDNAs was quantitated. The primer sets used in this study are listed in Table 1. The absolute copy number of each target cDNA in the samples was determined using the corresponding standard curve. The expression of HAS cDNA was normalized to that of GAPDH. Polymerase chain reaction specificity was confirmed using melting curve analysis, gel electrophoresis, and DNA sequencing.

**Table 1:** Primer pairs for quantitative reverse transcription polymerase chain reaction.

Gene name	Forward primer (5′–3′)	Reverse primer (5′–3′)	GenBank accession no.
HAS1	TAGGTGCTGTTGGAGGAGATGTGA	AAGCTCGCTCCACATTGAAGGCTA	XM_032893139.1
HAS2	ACTGGGCAGAAGCGTGGATTATGT	AACACCTCCAACCATCGGGTCTTCTT	XM_032890846.1
HAS3	TAGTGGATGGCAATCGCCAGGAAGAT	TTCACCCGCCTCATGGAAATTGCT	XM_032887382.1
GAPDH	GACAACTTTGGCATCGTGGA	ATGCAGGGATGATGTTCTGG	NM_017008.4

GAPDH, glyceraldehyde-3-phosphate dehydrogenase; HAS, hyaluronan synthase.

### 2.5. Histological study

#### 2.5.1. Sample collection

Rats were deeply anesthetized with pentobarbital and transcardially perfused with 50 mL of saline, followed by 500 mL of 10% formalin 7 days after intraperitoneal injection of VCR or saline (n = 8 in each group). The anterior tibial muscles of the lower legs were excised bilaterally, fixed in formalin for 2 hours, and incubated overnight in phosphate-buffered 30% sucrose. These tissues were embedded in an OCT compound (Sakura Finetek, Tokyo, Japan) and processed into 10-µm-thick frozen sections.

#### 2.5.2. Alcian blue staining

The sections were washed with distilled water and 3% acetic acid, respectively, for 2−3 minutes at room temperature, incubated with an Alcian blue solution (pH 2.5, Fujifilm Wako, Osaka, Japan) for 30 minutes, and washed with 3% acetic acid and distilled water for 5 minutes. Hyaluronic acid and mucus substances stained blue.

#### 2.5.3. Immunohistochemistry

Three isomers of HAS have been identified and highly conserved among mammalians (HAS1, HAS2, and HAS3), of which HAS2 plays a pivotal role. HAS2 knockout mice resulted in lethal in midgestation due to insufficient development of various organs, whereas other knockouts were not lethal.^9^ HAS2 also produces larger amount of HA faster than the others,^24^ which makes HAS2 important for wound repair. Therefore, we detected HAS2 immunoreactivity (ir) in the fascia. We also used hyaluronan-binding protein (HABP) due to its specificity to HA, whereas Alcian blue stains various types of acidic polysaccharides.

The frozen sections were washed with phosphate-buffered saline (PBS), blocked with 10% normal goat serum for 1 hour, incubated with anti-HAS2 (1:200, #sc-365263, Santa Cruz Laboratories, Santa Cruz, CA) or biotinylated HABP (1:250, Merck, Darmstadt, Germany) diluted in 1% normal goat serum incubation buffer overnight at 4°C. After repeated washes with PBS, the samples were incubated with the secondary antibody goat anti-mouse IgM-HRP (1:1000, SouthernBiotech, Birmingham, AL) for 2 hours or HRP-conjugated streptavidin (1:250, Proteintech, Rosemont, IL) for 30 minutes and washed in PBS. The reaction was then developed with 3,30-diaminobenzidine (DAB) and terminated with PBS. Cell quantification is expressed as the number of cells per field.

#### 2.5.4. Immunofluorescence

Stecco et al.^44^ showed that HA-rich cells are derived from a family of fibroblasts and suggested that these cells are called fasciacytes, a new class of fascia-associated cells, with S100A4 expression as their characteristic.

Double immunofluorescence staining was performed using the tyramide signal amplification kit (Thermo Fisher, Waltham, MA) to detect colocalization of HAS2 and S100A4 in the fascia. All sections were subjected to the following protocols: The tissues were washed in PBS for 3 minutes. After incubation with 0.1% H_2_O_2_ and 1% bovine serum albumin, frozen tissue sections were incubated with anti-HAS2 (mouse, 1:200, Santa Cruz Laboratories) and anti-S100A4 (rabbit, 1:100, # BS-3759R, Bioss, Woburn, MA) overnight at 4°C, followed by goat anti-mouse-DyLight 594 (1:500, Abcam, Cambridge, United Kingdom) and goat anti-rabbit-HRP (1:100, Promega, Fitchburg, WI) for 2 hours at room temperature. For S100A4 staining, sections were subsequently incubated with Alexa Fluor 488-conjugated tyramide (1:200, Thermo Fisher) for 10 minutes and mounted with ProLong Gold Antifade reagent with DAPI (Thermo Fisher). Images were acquired with a fluorescence microscope BZ-X700 (Keyence, Osaka, Japan) equipped with a 40× objective lens.

### 2.6. Statistical analysis

All data are expressed as mean ± standard error of the mean (SEM) or median with interquartile range. Statistical analysis was performed using the Mann–Whitney *U* test, Kruskal–Wallis test, and 2-way measurement analysis of variance followed by the Tukey post hoc test (GraphPad Prism 9, San Diego, CA). Statistical significance was set at *P* < 0.05.

## 3. Results

### 3.1. Vincristine-induced mechanical allodynia

#### 3.1.1. Mechanical threshold in the hind paw

**Figure 2.**
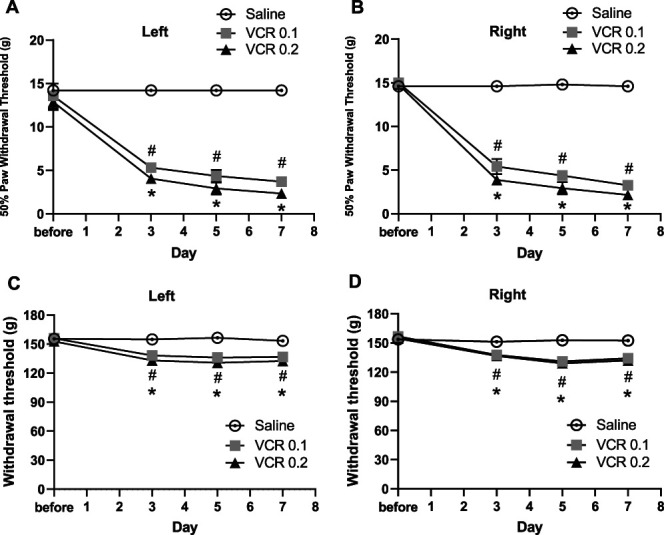
Changes in the mechanical thresholds in hind paw and tibialis anterior muscle. Mechanical thresholds were evaluated before and after intraperitoneal injection of VCR (0.1 or 0.2 mg/kg for a single injection). (A and B) Fifty percent paw withdrawal thresholds in the hind paws evaluated with von Frey filaments. (C and D) Mechanical thresholds in the tibialis anterior muscle evaluated with electronic von Frey aesthesiometer. The control group was given normal saline. Statistical analysis was performed using 2-way repeated-measures ANOVA followed by the Tukey test. Data are expressed as mean ± SEM. #*P* < 0.0001 VCR 0.1 (n = 5) vs NS group (n = 5) at each time point; *P < 0.0001 VCR 0.2 (n = 5) vs NS group at each time point. ANOVA, analysis of variance; NS, normal saline; VCR, vincristine.

Before administration, the baseline values of 50% PWT were not significantly different between the saline-treated and VCR-treated groups. Intraperitoneal injection of VCR at both doses resulted in a sustained reduction in 50% PWT after day 3 (Fig. 2A, B). Compared with the saline group, 50% PWTs in the bilateral hind paws decreased significantly from day 3 to day 7 (left: F_2,48_ = 182.6, *P* < 0.0001; right: F_2,48_ = 519.6, *P* < 0.0001 between the groups) in the VCR groups. There was no difference in 50% PWT between the 2 doses of VCR.

#### 3.1.2. Mechanical threshold in the tibialis anterior muscle

Before administration, the baseline values of mechanical withdrawal threshold were not significantly different between the saline-treated and VCR-treated groups. Vincristine at both doses resulted in a sustained reduction in withdrawal threshold after day 3 (Fig. 2C, D). Compared with the saline group, the withdrawal threshold was significantly decreased after day 3 to day 7 (left: F_2,48_ = 149.6, *P* < 0.0001; right: F_2,48_ = 41.03, *P* < 0.0001 between the groups). There was no difference in 50% PWT between the 2 doses of VCR.

### 3.2. Hyaluronic acid synthase mRNA expressions in the anterior tibial fascia

**Figure 3.**
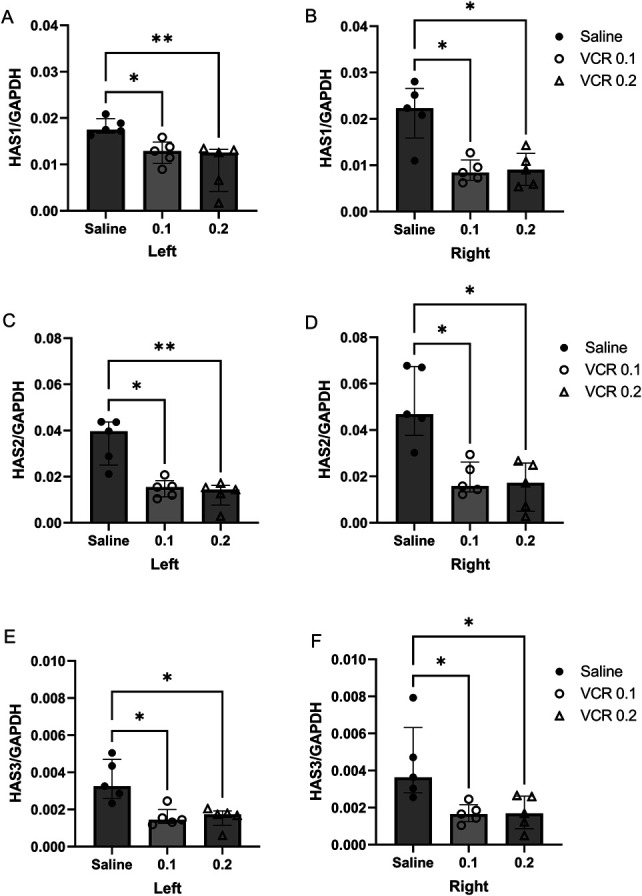
Quantitative RT-PCR of HASs in the bilateral anterior tibial fascial tissue. The expression of HAS1 (A and B), HAS2 (C and D), and HAS3 (E and F) is quantitated on day 7 after intraperitoneal injection of 0.1 mg/kg or 0.2 mg/kg of VCR. Control rats are injected with saline. The GAPDH expression is used as an internal standard. Data are presented relative to GAPDH and median with interquartile range. *P < 0.05 and **P < 0.01 vs saline group; n = 5. GAPDH, glyceraldehyde-3-phosphate dehydrogenase; HAS, hyaluronan synthase; RT-PCR, reverse transcription polymerase chain reaction; VCR, vincristine.

Changes in HAS expression after VCR administration are shown in Figure 3. HAS1, 2, and 3 mRNA were consistently expressed in the fascia. The relative expression of HAS2 was the highest among them, as previously reported,^9^ and the expression level of HAS3 was one order of magnitude lower than that of the others. The expression levels of HAS1, 2, and 3 in the VCR 0.1 mg/kg and VCR 0.2 mg/kg groups were significantly lower than in the saline group (*P* < 0.01). In this study, no dose dependence was observed.

### 3.3. Immunohistochemical analysis of HAS2 and hyaluronan-binding protein

**Figure 4.**
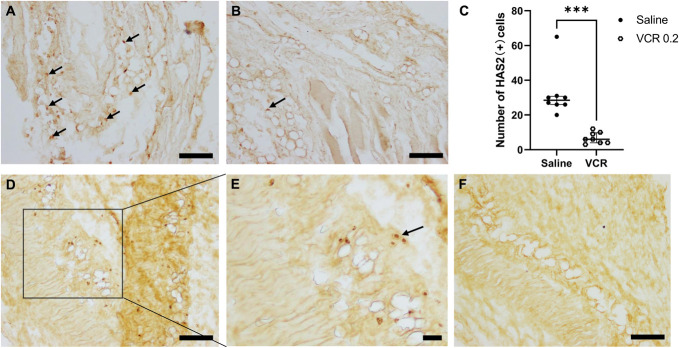
Immunohistochemistry of HAS2 and HABP. HAS2 immunoreactivity (ir) in the anterior tibial fascia of control (A) and VCR rats (B) on day 7. (C) Number of HAS2-ir cells was counted (n = 8 in each group). HA was stained by its specific binding protein, HABP, in the control (D and E) and VCR rats (F) on day 7. The bars in (C) are expressed as median and interquartile ranges. ***P < 0.001 compared with the control group by the Mann–Whitney test. Arrows indicate HAS2-ir cells (A and B) and cells with prominent nuclei and cytoplasm restricted to the perinuclear region (D and E). Scale bars: (A, B, D, F) = 100 μm and (E) = 50 μm. HA, hyaluronan; HABP, hyaluronan-binding protein; HAS, hyaluronan synthase; VCR, vincristine.

We then examined the localization and changes in HAS2 protein in fascial tissue in the control and VCR 0.2 mg/kg groups. As shown in Figure 4A, we found some rounded cells with strong expression of HAS2-ir in normal fascial samples. Seven days after VCR treatment, a significant loss of HAS2-ir cells was observed (Fig. 4B). As shown in Figure 4C, the number of HAS2-ir cells decreased from 28.5 (26−30.75) in the saline-treated group to 6.0 (4.0−9.75) (*P* < 0.001) in the VCR group. The HA in the ECM surrounding these cells was confirmed using biotinylated HABP. Hyaluronan-binding protein-immunoreactivity cells with prominent nuclei and cytoplasm restricted to the perinuclear region were more expressed in the control group, while they were difficult to detect in the VCR group (Fig. 4D–F).

### 3.4. Alcian blue staining

**Figure 5.**
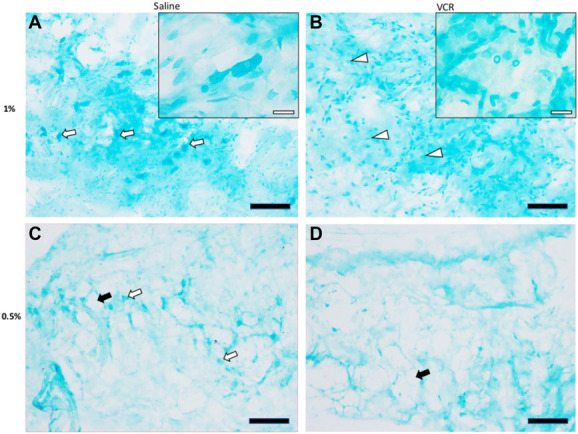
Alcian blue staining of the anterior tibial fascia. Acidic polysaccharides, including HA, are shown in blue with 1% Alcian blue solution in control (A) and VCR rats (B) on day 7. Extracellular matrix and small spindle-shaped cells were barely stained in 0.5% solution in control (C) and VCR rats (D). White arrows indicate fasciacytes, black arrows indicate adipocytes, and white arrowheads indicate damaged cyclic cells. Scale bars: black bars (A, B, C, D) = 100 μm; white bars (insets A and B) = 20 µm. HA, hyaluronic acid; VCR, vincristine.

Alcian blue staining at a concentration of 1% (Fig. 5A) showed that some bulky cells stained dark blue and many small elongated cells stained light blue, as well as acidic polysaccharides, including HA, in the extracellular space. Compared with the control group, we found a significant reduction in the number of larger dark blue cells in the VCR group (Fig. 5B). A previous study named these large dark cells as fasciacytes, which were different from fibroblasts in morphology, location, HA amount, and other markers.^44^ To avoid overstaining of cell bodies and polysaccharides, we used 0.5% Alcian blue solution, as shown in Figure 5C, D. Fibroblasts and other cells were rarely stained with 0.5% staining compared with 1% staining. Many large dark cells in the fascia were still observed in the control group, while fewer large dark cells were observed in the VCR group. These large dark cells are mainly distributed around the adipocytes. We also found some damaged cells in the VCR group, whose morphology became smaller and cyclic (Fig. 5B, inset).

### 3.5. Immunofluorescence of HAS2 and S100A4

**Figure 6.**
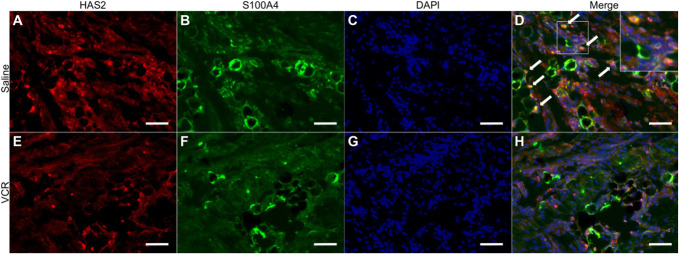
Double immunostaining of HAS2 and S100A4 in the rat fascia. HAS2 (red) and S100A4 (green) in the fascia of control (A–D) and VCR rats (E–H). The nuclei were stained with DAPI. White arrows indicate fasciacytes expressing HAS2 and S100A4. Scale bars: 50 μm. DAPI, 4,6-diamidino-2-phenylindole; HAS, hyaluronan synthase; VCR, vincristine.

Double immunostaining was used to characterize the HAS2-ir cells. The previous study suggested that the fibroblast-like cells with dense HA were distinguished from fibroblasts by the presence of S100A4.^44^ Colocalization of HAS2 and S100A4 was observed in the normal rat fascia (Fig. 6A-D). S100A4-ir was also found in adipocytes, as previously reported (Fig. 6B, F).^23^ We did not observe colocalization of the signals in the VCR group (Fig. 6E-H).

## 4. Discussion

In this study, we investigated the effect of the anticancer drug VCR on anterior tibial fascial tissue for HA production. We found a downregulation of HAS mRNA and reduction in the number of HAS2 highly positive cells in the fascia after VCR treatment, which could potentially lead to a worsening of chemotherapy-induced behavioral hypersensitivity. Patients with CIPN sometimes complain of difficulty moving their extremities and musculoskeletal pain. Although it is called “peripheral neuropathy,” the symptoms of CIPN may be partially due to the dysfunction of the fascia.

Many studies have shown that changes in HA homeostasis, a major component of ECM, are critical in relation to pain^2^ through multiple pathways. Hyaluronic acid has been widely used in clinical applications for pain treatment. For example, intra-articular injection of HA for osteoarthritis and targeted injection for plantar fasciopathy have obtained positive effects.^26,39^ In the HAS gene family, HAS1 and HAS2 produce longer HA chains, resulting in higher-molecular-weight HA.^24^ Several studies have reported that high-molecular-weight HA attenuates the mechanical hyperalgesia induced by inflammatory mediators in rats,^5^ modulates the opening of the TRPV1 channel, and reverses the sensitization of peripheral nociceptors.^8^ Furthermore, there is much evidence that receptor activation strongly depends on the viscoelasticity of tissues; therefore, HA is one of the most important determinants of tissue viscoelasticity, and its alteration can modify receptor activation within the fascia.^45,46^ Only 1 study reported an association between HA and chemotherapy, in which HA was positively correlated with the water content in the skin of patients with breast cancer after chemotherapy compared with those without chemotherapy.^25^ In this study, we found that the expression of HAS1 and 2 was significantly downregulated, which may affect the synthesis of high-molecular-weight HA and thus worsen the course of nociceptive hypersensitivity. Hyaluronic acid is also often used in dermatology, where it has been shown to play a key role in the inflammatory, proliferative, and remodeling phases of the wound healing process of the skin.^28,37^

Although fascia has been overlooked in pain research, several studies have focused on fascial neural networks. Fascia has been well documented to be rich in innervation, the superficial fascia shares mechanical and thermal receptors with the skin, and these innervations are interspersed with adipocytes.^3,15,49^ A previous study reported an important breakthrough in research on the origin of HA in the fascia, a new type of cell termed fasciacytes, which is committed to producing the HA-rich ECM found in connective tissue.^44^ This study suggests that fibroblast-like cells with dense HA, called fasciacytes, affect fascial gliding because these cells are primarily located at the borders of the fibrous fascial sublayer. If fascial tissue is lost or its density is altered, this can lead to a fascial sliding defect and impair the entire function of the tissue, causing pain^7,41^ by stimulating the nociceptors. Our results reported the effect of chemotherapeutic agents on HAS2 highly positive cells and fascial damage, which may have impaired the ability of the fascia to slide after chemotherapy treatment. This is a significant concern for the health of patients undergoing chemotherapy.

Fasciacytes are also likely to be located in the fascial zone, an area with a greater degree of innervation (more nerve endings and Pacini and Ruffini vesicles).^42,43^ Recently, the fascia has been considered a proprioceptive organ and can be altered by stress, trauma, overuse, and surgery.^15,47,52^ Our results show that HAS2 highly positive cells are also abundantly distributed around adipocytes, but we do not understand how these cells interact with nerves and adipocytes. In addition, positive HA staining has been reported in the perivascular and perineural tissues of the fascia.^27,32^ Previous studies have reported the anatomical and morphological characteristics of fasciacytes, in normal tissues; however, the pathological impact of fasciacytes remains unclear. We speculate that those cells may produce enough HA to provide a stable microenvironment for nerves, thus protecting them from additional stimulation. In addition, we also found that VCR disrupted fibroblasts to some extent, causing changes in cell morphology, which could make some changes in the structure of the fascia. Therefore, further exploration is necessary.

This study had several limitations. First, our results are phenomena observed in CIPN model rats and did not show direct involvement of HA and pain behavior. Because the role of HA in CIPN has not been reported previously, further studies are needed to clarify the relationship. Topical supplementation with HA is used clinically for some orthopedic diseases; however, it cannot be applied to CIPN, whose symptoms manifest systemically. Cannabinoid receptor 2 agonists have been reported to promote HA production in fasciacytes.^16^ Cannabidiol, a nonaddictive cannabis drug approved by the U.S. Food and Drug Administration, has been studied in many fields and is expected to play a role in the treatment of CIPN pain.^29,31,50,51^ Therefore, we speculated that the effect of cannabinoid agonists on CIPN is partially ascribed to HA production. Another limitation was that we could not quantify the amount of HA in the fascia. However, because HASs are unique sources of HA, we believe that downregulation of HASs leads to a reduction in HA. Furthermore, the total amount of HA is not necessarily important for pain reduction because low-molecular-weight HA has been reported to induce hyperalgesia, which was attenuated by high-molecular-weight HA.^17^ Because HAS2 produces longer HA chains, decreased HAS2 will considerably affect pain sensation.

## 5. Conclusion

In summary, the data from the present study indicate that mRNA expression and protein levels of HAS were decreased in the fascia of CIPN model rats. The affected HA production in the fascia, as well as the damage to the fascia, could be one of the possible causes of the decreased pain threshold in CIPN model rats. This study suggests new therapeutic and research targets for the treatment of chemotherapy-induced “peripheral neuropathy.”

## Disclosures

The authors have no conflict of interest to declare.
